# “Autoimmune(-Like)” Drug and Herb Induced Liver Injury: New Insights into Molecular Pathogenesis

**DOI:** 10.3390/ijms18091954

**Published:** 2017-09-12

**Authors:** Marcial Sebode, Lisa Schulz, Ansgar W. Lohse

**Affiliations:** Department of Medicine, University Medical Centre Hamburg-Eppendorf, 20246 Hamburg, Germany; l.schulz@uke.de (L.S.); alohse@uke.de (A.W.L.)

**Keywords:** idiosyncratic, drug-induced liver injury, autoimmune hepatitis, herbal and dietary supplements, herbs, autoimmune-like drug induced liver injury

## Abstract

Idiosyncratic drug-induced liver injury (DILI) and hepatic injury due to herbal and dietary supplements (HDS) can adapt clinical characteristics of autoimmune hepatitis (AIH), such as the appearance of autoantibodies and infiltration of the liver by immune competent cells. To describe these cases of DILI/HDS, the poorly-defined term “autoimmune(-like)” DILI/HDS came up. It is uncertain if these cases represent a subgroup of DILI/HDS with distinct pathomechanistic and prognostic features different from “classical” DILI/HDS. Besides, due to the overlap of clinical characteristics of “immune-mediated” DILI/HDS and AIH, both entities are not easy to differentiate. However, the demarcation is important, especially with regard to treatment: AIH requires long-term, mostly lifelong immunosuppression, whereas DILI/HDS does not. Only through exact diagnostic evaluation, exclusion of differential diagnoses and prolonged follow-up can the correct diagnosis reliably be made. Molecular mechanisms have not been analysed for the subgroup of “autoimmune(-like)” DILI/HDS yet. However, several pathogenetic checkpoints of DILI/HDS in general and AIH are shared. An analysis of these shared mechanisms might hint at relevant molecular processes of “autoimmune(-like)” DILI/HDS.

## 1. Introduction

In drug-induced liver injury and hepatic injury due to herbal and dietary supplements (DILI/HDS), the triggering event has been identified by definition. The intake of a drug, herb or supplement leads to a usually acute hepatitis. A subgroup of idiosyncratic DILI/HDS cases show features of autoimmunity such as the presence of autoantibodies and pronounced hepatic infiltration of immune competent cells. To describe these cases of DILI/HDS, the term “autoimmune(-like)” DILI/HDS has been applied. This subgroup of DILI/HDS resembles autoimmune hepatitis (AIH). In contrast to DILI/HDS, the triggering event for AIH is elusive and the main antigen leading to chronic inflammation of the liver is unknown for the majority of AIH patients. The clinical similarities of “autoimmune(-like)” DILI/HDS and AIH have led to several questions: how can “immune-mediated” DILI/HDS be differentiated reliably from AIH? This is relevant for treatment, but also for previous and future pathogenetic studies: is the clinical diagnosis sufficiently certain to assure that the experimental results represent the suspected disease? What are the shared molecular mechanisms of both entities? Molecular mechanisms have not been analysed for the subgroup of “autoimmune(-like)” DILI/HDS yet. That is why this review deals with pathogenetic mechanisms of DILI/HDS in general and of AIH. Insights into these molecular processes may hint at relevant pathogenetic mechanisms of “autoimmune(-like)” DILI/HDS. Due to the similarities between idiosyncratic DILI/HDS and AIH, the general clinical context of both entities must, firstly, be clarified, before the respective molecular pathogenesis can be illustrated.

## 2. Clinical Context of Drug-Induced Liver Injury and Hepatic Injury due to Herbal and Dietary Supplements (DILI/HDS) and Autoimmune Hepatitis (AIH)

### 2.1. Drug-Induced Liver Injury and Hepatic Injury Due to Herbal and Dietary Supplements

Drug-induced liver injury (DILI) is characterized by a broad spectrum of clinical appearances [1]. Manifestations of DILI range from mild elevation of liver enzymes to acute liver failure (ALF). Biochemical patterns of DILI can be hepatocellular, cholestatic or mixed. The histological picture is diverse, comprising steatosis, infiltration of immune competent cells, necrosis, cholestasis, vanishing bile duct syndrome, sinusoidal obstruction syndrome and others [2,3]. The leading pharmaceutical class causing non-acetaminophen DILI consists of antimicrobials [4]. Liver injury due to herbal and dietary supplements (HDS) incorporate a variety of agents, primarily multi-ingredient nutritional or dietary supplements, body building products with anabolic steroids and single as well as multiple herbal products [5]. The incidence of DILI is about 14–19 per 100,000 inhabitants in population-based studies [6,7]. The proportion of HDS cases causing hepatotoxicity has increased in the United States from 7% in 2004–2005 to 20% in 2013–2014 according to the Drug Induced Liver Injury Network (DILIN) [5,8]. DILI and liver injury due to HDS are responsible for more than 50 % of ALF cases [9,10].

DILI is grouped into idiosyncratic and intrinsic forms [11]. This classification stems from clinical observations and probably reflects different molecular mechanisms. Intrinsic forms of DILI are mainly represented by acetaminophen (APAP, acetyl-para-aminophenol) that is characterized by a clear dosage-dependency, a predictable clinical course and a more direct hepatotoxic pathogenesis. In brief, the highly reactive toxic APAP-metabolite *N*-acetyl-*p*-benzoquinoneimine (NAPQI) accumulates in the liver after the depletion of glutathione and leads to hepatic necrosis. In addition, the immune system also takes part in APAP pathogenesis [12]. This supports the assumption that DILI/HDS cannot be reduced to one single molecular mechanism causing liver damage.

In contrast to intrinsic forms, idiosyncratic DILI is less predictable and occurs only in a minority of patients exposed to a drug. Idiosyncratic DILI can be subdivided into allergic and non-allergic forms [11]. The allergic subtype is accompanied by typical features of allergy such as rash, eosinophilia, fever and short latency after drug exposure. One of its severest forms is the DRESS-syndrome (Drug rash with eosinophilia and systemic symptoms) [13]. DRESS is caused by phenytoin, carbamazepine, minocycline and a variety of other drugs. Non-allergic subtypes of idiosyncratic DILI are characterized by the absence of clinical signs of allergy and have a longer latency instead. The concept of dosage-independency has been revised for idiosyncratic DILI: according to recent studies, the risk of DILI is increased if a minimal threshold of dosage is exceeded [14].

The diagnosis of DILI/HDS is difficult to make. It is supported by scores of causality assessment, e.g., RUCAM (Roussel Uclaf Causality Assessment Method) [15,16,17]. Features of RUCAM constitute, among others, the exclusion of acute viral hepatitis, AIH and other liver diseases before the diagnosis of DILI can be made. However, concrete diagnostic criteria on how to differentiate DILI/HDS from AIH are missing in the current causality scores for DILI. 

Treatment of DILI/HDS starts with the correct identification and prompt stopping of the causative agent. In severe cases of acute liver failure, liver transplantation impends [9,10]. For “immune-mediated” forms of DILI, immunosuppressive therapy might seem to be a logical option. However, the efficacy of steroids for DILI/HDS is controversial and based on little evidence. Two older studies analysed the usage of steroids in patients with acute liver failure due to different entities like fulminant viral hepatitis or DILI [18,19]. They were unable to show a survival benefit for DILI cases under steroids. These older studies bear the risk of other causes of acute liver injury having been missed, as they were not on the agenda at that time. For example, autochthonous Hepatitis E infection as a cause of acute hepatitis has emerged in Europe and the USA in the last 20 years and must be excluded before diagnosing DILI/HDS today [20,21]. Another study advocating against the use of steroids for DILI has been performed in the setting of ALF [22]. In this retrospective study, steroid treatment was compared to the spontaneous course of a mixed group of AIH, DILI and indeterminate cases of “immune-mediated” liver injury. Steroid usage was not associated with improved overall survival. All cases were severely ill with a median MELD (Model of end-stage liver disease) score above 30 points. This may reflect that the time point of return was probably missed and liver damage was already too advanced. Besides, only 16 DILI cases under steroids have been analysed in this study. Small case series indicate a benefit of steroids for the clinical course of DILI. Patients with minocycline- and nitrofurantoin-induced hepatitis, both drugs typically leading to “immune-mediated” forms of DILI, improved under steroids [23]. In another case series of DILI, steroid treatment led to a more rapid decline of liver enzymes in comparison to spontaneous courses [24]. Next to immunosuppression, an alternative therapeutic approach for DILI lies in restoring hepatic glutathione levels by *N*-acetylcysteine (NAC) and thereby diminishing reactive metabolites. In a prospective, double-blind study, NAC improved transplant-free survival in early stages of non-acetaminophen ALF [25]. Again, the study population contained a mixture of entities (DILI, AIH, hepatitis B virus infection and indeterminate causes). Patients with advanced hepatic encephalopathy did not benefit from NAC and required liver transplantation. Overall, the evidence for medical treatment of DILI/HDS is poor and clear recommendations cannot be given. For severe cases, liver transplantation has to be considered in time. Prompt identification and stopping of the causative agent is still the mainstay of DILI/HDS treatment.

### 2.2. Autoimmune Hepatitis

Autoimmune hepatitis (AIH) is a chronic inflammatory liver disease with unknown aetiology [26]. It is characterized by typical, but not disease-specific features such as the presence of autoantibodies, elevation of IgG/gammaglobulins, exclusion of viral hepatitis and a typical histological appearance. These features are included in the simplified AIH score [27]. The previously proposed revised IAIHG (International Autoimmune Hepatitis Group) score comprises more features than the simplified AIH score, but is less suitable for daily practice [28,29]. The exclusion of DILI/HDS is a feature of the revised IAIHG score but parameters on how to exclude DILI are absent. The simplified AIH score does not contain the exclusion of DILI/HDS as a criterion and has not been validated in patients with DILI/HDS yet. The characteristic histological pattern of AIH is an interface hepatitis of infiltrating lymphocytes exceeding the borders of the portal tract [27]. Although a considerable overlap of histological criteria exists between AIH and DILI/HDS, particular patterns may favour one diagnosis over the other [30]. Plasma cells, rosette formation of hepatocytes and emperipolesis (engulfment of inflammatory cells by hepatocytes) are more prevalent in liver biopsies of AIH patients. Nonetheless, these histological patterns are still in need of validation.

The detection of autoantibodies such as ANA (anti-nuclear antibodies), anti-SMA (antibodies directed against smooth muscle antigen), anti-LKM (antibodies directed against liver-kidney microsomes), anti-SLA/LP (antibodies directed against soluble liver antigen/liver-pancreas antigen) and/or anti-LC1 (antibodies directed against liver cytosol) supports the diagnosis of AIH [31]. Diagnostic scores of AIH differentiate between very low and higher titres of antibodies (>1:40 vs. >1:80) [27,29]. It is unclear whether very high titres (e.g., 1:1280) have an even higher diagnostic specificity. The specificity for AIH varies for each of these antibodies: anti-SLA/LP are highly specific for AIH [32]. However, they are present in only 10–15% of all AIH patients. In up to 10% of AIH patients, anti-SLA/LP are the solely detectable antibodies [33]. Anti-SLA/LP can only be detected by ELISA (Enzyme-linked Immunosorbent Assay) or immunoblot and not by immunofluorescence [31]. Anti-LKM and anti-LC-1 are also specific for AIH, though to a lesser degree than anti-SLA/LP [34,35]. Anti-LKM are present in up to 5% of AIH patients and they are associated with early age disease manifestation [36]. Anti-SMA can be grouped into antibodies directed against actin, tubulin or intermediate filaments. While those directed against tubulin and intermediate filaments can be found in a variety of liver diseases, anti-SMA showing reactivity against F-Actin seem to be more specific for AIH [37,38]. ANA are not specific for AIH and occur in other liver diseases such as primary sclerosing cholangitis (PSC) [39]. In the setting of ALF, unspecific elevation of autoantibodies can be detected in various liver diseases like DILI or acute viral hepatitis [40]. These clinical observations support that the presence of most autoantibodies is not pathognomonic for AIH. In addition to being unspecific, autoantibodies are not essential for the diagnosis of AIH, as retrospective analyses have shown that AIH patients can be negative for all the above-mentioned autoantibodies [41]. AIH is classified by the pattern of autoantibodies: positivity for ANA and anti-SMA refers to type 1 AIH, whereas the detection of anti-LKM and anti-LC-1 refers to type 2 AIH [31]. Whether the presence of anti-SLA/LP or anti-SMA is associated with a worse prognosis of AIH, is still a matter of debate [42,43].

The respective antigens that are recognized by autoantibodies are only partly known for AIH: anti-SLA/LP are directed against SEPSECS (*O*-phosphoseryl-tRNA:selenocysteinyl-tRNA synthase), an enzyme of selenocysteine formation [44]. Its epitopes are recognized by both the humoral and the T cell-mediated immune response [45]. This points to a relevant pathogenetic role of SEPSECS for AIH. Anti-LKM target epitopes of the cytochrome P450 (CYP) 2D6 in AIH patients [46,47,48]. Anti-LC1 are directed against formiminotransferase cyclodeaminase (FTCD), an enzyme involved in both histidine and folate metabolism [49,50]. The antigens that are targeted by ANA in AIH are widely unknown [51].

**Figure 1:**
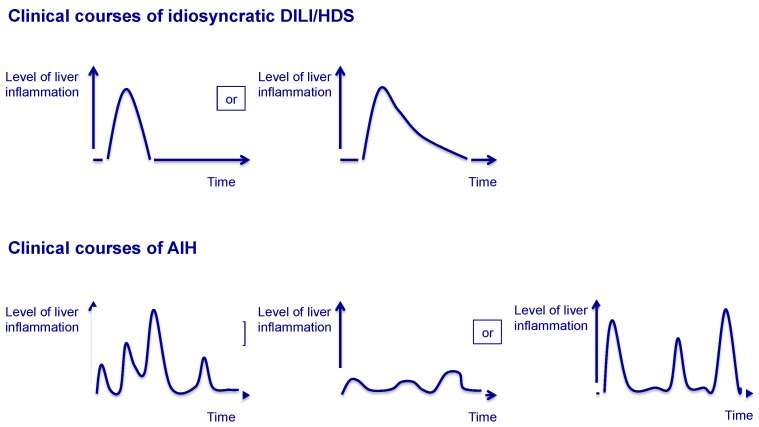
Different clinical courses of herbal and dietary supplements (DILI/HDS) and Autoimmune hepatitis (AIH).

The clinical course of AIH is chronic and fluctuating. Episodes of spontaneous remission can occur, but it is unpredictable whether inflammation returns, even in form of ALF. The chronic course of AIH helps to differentiate AIH from DILI/HDS (Figure 1). Treatment of AIH is based on long-term, in most cases lifelong immunosuppression [52]. To induce remission, prednis(ol)one is applied. With a delay of 1–2 weeks after initiation of steroid treatment, azathioprine is introduced to maintain remission in the long run. Usually, transaminases and IgG/gammaglobulins decline after introduction of prednis(ol)one. Steroids are then gradually reduced and, in most cases, azathioprine-monotherapy can control liver inflammation. The aim of AIH treatment is complete normalization of transaminases and IgG/gammaglobulins, both of which are relatively good surrogate markers for the absence of intrahepatic inflammation. An increase in transaminases and/or IgG/gammaglobulins in the process of dosage finding hints to recurring AIH activity and requires adaptation of therapy. Gradual discontinuation of immunosuppression can be considered for AIH after several years of remission under treatment. However, the majority of AIH patients will relapse after cessation of immunosuppression [53]. Relapse can occur with a delay of a couple of years after discontinuation of immunosuppression. In a retrospective analysis, about 60% of AIH patients required reintroduction of immunosuppression one year after complete withdrawal of immunosuppressive treatment, increasing to 80% after 3 years [53]. These clinical observations support the chronic character of AIH with intermittent episodes of low or absent inflammatory activity (Figure 1). The pathogenetic background for this fluctuating course of AIH is unknown.

In summary, DILI/HDS and AIH have overlapping diagnostic features. Up to today, there is no diagnostic test to reliably differentiate DILI/HDS from AIH in the setting of acute hepatitis. In case of uncertainty, a steroid pulse therapy and close follow-up of transaminases and IgG/gammaglobulins during weaning and after cessation of steroids will uncover the correct diagnosis: AIH will relapse and transaminases and/or IgG/gammaglobulins will rise again. In DILI/HDS, liver enzymes will stay normal if the causative drug has been identified and stopped.

### 2.3. Clinical Scenarios Involving Both DILI/HDS and AIH

Additional to classical courses of AIH or DILI/HDS, variant forms mimicking or involving the other entity can occur [54]. These cases are hard to define since there is no consensus on the nomenclature and aetiology. Several scenarios are possible (Table 1). These scenarios may help to illustrate the uncertainties the clinician is confronted with when he has to differentiate DILI/HDS from AIH.

The first scenario involves AIH patients in remission under low-dose therapy who develop DILI/HDS from the intake of a new drug or supplement. This scenario must be distinguished from an aggressive, relapsing course of AIH that is independent from the intake of a new drug. Both scenarios will become evident through elevated liver enzymes. In the case of aggressive AIH, levels of IgG/gammaglobulins mostly parallel the rise of transaminases. In addition to IgG/gammaglobulins, the exact drug history of the last 6–12 months, including herbs and supplements, helps to make the correct diagnosis. Every new drug or agent that has been started must be checked for its potential to cause DILI/HDS. Therapeutically, DILI/HDS on top of AIH can be controlled by cessation of the causative drug. For more severe cases, a short-term pulse therapy with steroids can be considered. An aggressive form of AIH needs intensification of immunosuppression or second-line therapy.

In a second scenario, an episode of DILI/HDS triggers chronic AIH. This constellation has been proposed by case reports [55]. However, causality between drug intake and manifestation of chronic AIH cannot be proven thus far. Therefore, this scenario seems unlikely. More likely is a scenario of pre-existent AIH and coincidental drug-exposure or, indeed, coincidental DILI/HDS as described in the first scenario.

In a third scenario, a second episode of DILI/HDS mimics a relapsing form of AIH. However, Lucena et al. showed that a second episode of DILI/HDS in response to a structural related drug or agent is rare and happens only in about 1% of all DILI/HDS cases [56].

A fourth scenario involves an unrecognized and continuous drug intake leading to chronic hepatitis and liver damage [57,58]. Such an unrecognized chronic DILI/HDS can mimic AIH and is probably more frequent in HDS than in DILI: the prevalence of underreporting and trivialization is higher for herbs and supplements than for drugs. In consequence, identification of the causative agent is hindered even more. All in all, the scenario of chronic DILI/HDS is less likely to occur than AIH. Chronic course and high-grade fibrosis or cirrhosis still favour the diagnosis of AIH. Up to 20–30% of AIH patients are diagnosed in the stage of cirrhosis [59,60,61].

In a fifth scenario, cases of idiosyncratic DILI/HDS show overlapping clinical characteristics of AIH. For this subgroup of DILI/HDS, the descriptive terms “autoimmune(-like)” and “immune-mediated” DILI/HDS came up. This subgroup has not been defined precisely. Therefore, such a subclassification of DILI remains artificial and is solely descriptive without scientific basis. However, the term has been used for cases of acute hepatocellular injury with elevated autoantibodies, female predominance and histological features resembling AIH that are timely associated with a drug, herb or supplement intake as a trigger. The relevant difference to AIH is that cases of “autoimmune(-like)” DILI do not recur after discontinuation of immunosuppression [23]. Some case reports have applied the revised IAIHG score or the simplified AIH score in order to make the diagnosis of “autoimmune(-like)” DILI/HDS. Yet these scores have not been developed for this purpose. Whether cases of “autoimmune(-like)” DILI/HDS represent a subgroup of all DILI/HDS with a different molecular pathogenesis or clinical course is unknown. Again, coincidence without any causal relationship must be considered: the true nature of designated “autoimmune(-like)” DILI/HDS could be that of classical AIH with the intake of a drug just being coincidental. The following drugs are more frequently associated with “autoimmune(-like)” DILI/HDS: nitrofurantoin, minocycline, diclofenac, methyldopa and tumor necrosis factor-α (TNFα) inhibitors [62,63,64,65]. It is tempting to assume that patients with “autoimmune(-like)” DILI/HDS would benefit from immunosuppressive treatment but there is still not enough evidence to recommend this line of treatment.

In summary, there is a considerable overlap of the clinical characteristics of idiosyncratic DILI/HDS and AIH, especially in cases of “autoimmune(-like)”, “immune-mediated” DILI/HDS. Interpretation and definition of these cases are highly controversial and diagnostic uncertainties are the consequence. But the correct diagnosis is of importance: in contrast to acute DILI/HDS, chronic AIH needs long-term immunosuppression. DILI/HDS misdiagnosed as AIH leads to unnecessary long-term immunosuppression with potential side effects like non-melanoma skin cancer or lymphoma. In cases of AIH misdiagnosed as DILI/HDS, long-term immunosuppression is not started and a second, potentially fulminant relapse of AIH impends. Only few characteristics help to differentiate AIH from DILI/HDS. During withdrawal or after complete cessation of steroids, AIH will relapse in almost all cases [53]. In contrast, DILI/HDS will not relapse if the causative agent has been stopped. Physicians need to be aware of the respective clinical courses (Figure 1) and monitor liver enzymes closely. Due to spontaneous episodes of remission, a relapse of AIH can occur several months or even years after first presentation. Further features which help to differentiate DILI/HDS from AIH are the stage of liver fibrosis and clinical signs of hypersensitivity: DILI/HDS rarely causes liver cirrhosis, whilst symptoms like fever, rash and eosinophilia are unusual in AIH [52].

## 3. Molecular Mechanisms of DILI/HDS and AIH

Additional to clinical characteristics, DILI/HDS in general and AIH share several molecular checkpoints in their respective pathogenesis (Table 2). For the subgroup of “autoimmune(-like)” DILI/HDS, molecular mechanisms have not yet been analysed. However, pathogenetic insights into DILI/HDS in general and AIH might hint at molecular processes that are ongoing in “autoimmune(-like)” DILI/HDS.

### 3.1. Genetic Background

The strongest genetic association of both DILI/HDS and AIH is located within the major histocompatibility complex (MHC)/human leucocyte antigen (HLA) region. This genetic association underscores the relevance of antigen presentation by MHC molecules to T cells for the pathogenesis of both entities. In Europe and North America, susceptibility for type 1 AIH is increased by the alleles DRB1*03:01 and DRB1*04:01 encoding HLA-DR3 and HLA–DR4 [66,67]. HLA-DR3 or HLA-DR4 are features of the revised IAIHG score favouring the diagnosis of AIH [29]. A recent genome-wide association study (GWAS) in a population of Dutch and German patients confirmed that these HLA genotypes convey susceptibility to AIH [68]. Risk HLA alleles vary geographically, as, for example, HLA-DRB1*04:05 is associated with susceptibility to AIH in Japan [69]. This might reflect that not a single antigen is presented by HLA molecules to effector cells in AIH, but instead several antigens which vary worldwide.

HLA variants that have been associated with susceptibility to AIH are different from those for DILI/HDS. HLA risk alleles have been analysed in DILI caused by flucloxacillin, amoxicillin-clavulanate, isoniazid, rifampicin, lapatinib, ticlopidine, terbinafine and nevirapine [70,71]. As an example, HLA-B*57:01 is consistently associated with flucloxacillin-induced DILI [72]. In amoxicillin-clavulanate-induced DILI, various HLA-associations have been detected, such as the HLA-class II alleles DRB1*15:01 and DQB1*06:02 and the HLA-class I allele HLA-A*02:01 [73]. Only few HLA variants are associated with adverse reactions of more than one drug. HLA-B*57:01 is associated with flucloxacillin-induced DILI and abacavir-induced skin hypersensitivity [72,74]. Even protective HLA alleles lowering the risk for DILI/HDS have been identified. HLA-DRB1*15:01 lowers susceptibility to flucloxacillin-induced DILI and HLA-DRB1*07:01 is protective for amoxicillin-clavulanate-induced DILI [70]. However, a recent GWAS for DILI including about 20 drugs (cases of flucloxacillin and amoxicillin-clavulanate DILI were excluded) confirmed only HLA-A*33:01 as a risk allele for terbinafine-induced DILI [75]. Previously reported HLA-associations for other drugs were not confirmed by this study.

Immunological genes outside the HLA-region have been associated with susceptibility to DILI/HDS and AIH. In North American AIH patients, susceptibility to AIH is increased by genetic variants of regulatory and pro-inflammatory receptors and molecules like CTLA-4 (Cytotoxic T lymphocyte antigen 4) and TNFα [76,77]. Yet in other countries these associations could not be confirmed. GWAS has identified variants of SH2B3 (Scr homology 2 adaptor protein 3, Lnk) and CARD10 (caspase recruitment domain 10) as likely risk factors for AIH [68]. Among other functions, SH2B3 is a negative regulator of T cell activation [78]. CARD10 is a scaffold protein participating in the signalling pathways of apoptosis and is expressed in various cell types, including hepatocytes [79]. In DILI/HDS, polymorphisms resulting in low interleukin-(IL-)10 and high IL-4 expression, thereby favouring a Th2-mediated immune reaction, have been associated with diclofenac-induced liver injury [80]. Aside from immunological genes, genetic polymorphisms of drug metabolism have been associated with susceptibility to DILI/HDS [81]. Genetic variants of impaired phase I and II biotransformation [82,83,84] and dysregulated mitochondrial protection against oxidative stress [85,86,87,88] increase the risk for DILI/HDS.

### 3.2. Neoantigens, Antigen Presentation and Triggering Events

The liver is naturally exposed to a diversity of antigens: it is one of the first organs to come into contact with ingested food and drugs. Neoantigen formation is a result of metabolized and processed antigens and takes place in the liver. Haptenization is part of this process: small molecules only lead to an immunological response when they are attached to bigger, but harmless carrier proteins.

**Figure 2:**
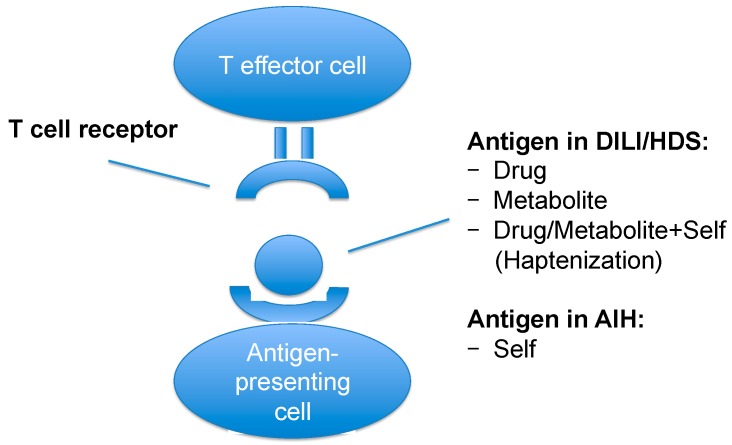
Antigen presentation in DILI/HDS and AIH.

In DILI/HDS, the immune reaction can be directed against the drug, the metabolite(s) of the drug or against the drug/metabolite attached to a self-protein (hapten). Though the main antigen has not been identified for AIH, it is assumed that its pathogenesis is antigen-specific and directed against a self-protein. In this case, DILI/HDS and AIH share primarily the same pathomechanism, the only differences being the kind of the antigen (Figure 2) and the duration of antigen exposure: in AIH a self-antigen is probably presented to the immune system in a constant manner. Alternatively, the immune reaction in AIH could be directed not against a self-antigen, but against an environmental, nutritional or bacterial antigen (or their metabolites). Thus, both entities would share even more pathomechanistic aspects.

Hapten-formation has been detected in flucloxacillin-induced DILI: conjugates of flucloxacillin and albumin are critical for the generation of flucloxacillin-specific T cells [89]. Haptenization and antigen-processing can result in molecular mimicry which has been proposed as a pathogenetic mechanism for various autoimmune diseases. Molecular mimicry is based on cross-reactivity of the immune system against harmless self-antigens with structural homology to exogenous pathogens. It has been proposed to be involved in the pathogenesis of type 2 AIH due to a sequence homology between peptides of the hepatitis C virus and epitopes of CYP 2D6 [90].

The liver provides a microenvironment that induces tolerance, even to self-antigens that are naturally located outside the liver. In a mouse model of multiple sclerosis (MS), hepatic expression of myelin basic protein, one of the main neuronal autoantigens, protected against the neurological phenotype [91]. In this model, tolerance was not induced whenever the antigen was expressed in other organs than the liver. Hepatic antigen-presenting cells (APC) determine the tolerogenic properties of the liver. They comprise different cell types such as dendritic cells (DC), liver sinusoidal endothelial cells (LSEC), Kupffer cells and hepatic stellate cells. Hepatic APC express HLA-molecules for antigen-presentation, co-stimulatory and co-inhibitory molecules, cytokines and chemokine receptors to fine-tune the immune response [92]. Every type of APC has its unique properties to adapt the immune response. As an example, LSEC can actively suppress pro-inflammatory cytokine production of Th1 and Th17 cells in vitro. In contrast, DC or Kupffer cells are not able to control inflammation to such an extent [93]. For DILI/HDS, it is unknown on which cell type drug-derived antigens are presented to the immune system and for how long. In AIH, the main antigen is unknown for the majority of patients and, therefore, the kind of antigen presentation is elusive, too. In type 2 AIH, CYP 2D6, the antigen against which anti-LKM are directed, has been detected on the surface of hepatocytes [93].

To trigger hepatic inflammation, antigen presentation may not be sufficient. Reactive metabolites of various drugs form haptens, but do not consequently lead to DILI [94]. A viral infection as a second hit has repeatedly been proposed as being involved in the induction of AIH, but has not been demonstrated convincingly [95]. Such a second hit alters the initial steps of antigen presentation: hepatocytes constitutively express HLA class I molecules, but under inflammatory conditions, HLA class II molecules are additionally expressed [96]. AIH mouse models have supported the expression of a foreign antigen in the liver being insufficient to break immune tolerance: additional pro-inflammatory stimuli are necessary to activate the immune system and promote an antigen-specific immune response [97].

### 3.3. Metabolism

Impaired metabolism of drugs, herbs or supplements causes liver damage by the toxic effect of reactive metabolites and danger signals. Metabolism starts with basic pharmacological characteristics of the agent: hepatobiliary excretion, lipophilicity and a minimal dosage of a drug are associated with the risk for DILI/HDS [14,98,99,100]. Individual co-factors such as age and sex alter CYP-mediated metabolism and drug excretion, thereby increasing the susceptibility to DILI/HDS [101,102,103]. Nutritional status and co-medication of the patient additionally influence drug metabolism. Deficiency of l-carnetine promotes valproate-induced DILI [104]. Alcohol consumption results in the induction of CYP 2E1 and increases NAPQI-formation. Thereby, APAP-induced liver injury is aggravated [105]. Some of the individual co-factors increasing susceptibility to DILI/HDS refer to the inflammatory stress hypothesis. It states that acute inflammatory episodes occurring during drug exposure can sensitize an individual to DILI/HDS and may decrease the threshold for hepatotoxicity [106]. The conditions leading to inflammatory stress are diverse, including infections, alterations in diet, alcohol consumption, surgical trauma and others [107]. Murine models showed that a number of drugs causing idiosyncratic DILI, such as amiodarone or diclofenac, are hepatotoxic in a condition of modest inflammation, but do not cause liver injury in control animals without ongoing inflammation [108,109].

Drug metabolism includes two pathways: phase I of biotransformation includes oxidative, reductive, and hydrolytic reactions resulting in reactive metabolite formation. For some drugs, the pharmaceutical effect is based on reactive metabolite formation, whereas for others it results from metabolite inactivation. As a side effect, intermediate reactive metabolites such as reactive oxygen species (ROS) can covalently bind proteins and form neoantigens [110]. Alternatively, ROS deplete protective antioxidants like glutathione leading to oxidative stress. Mitochondrial dysfunction due to inhibition of the respiratory chain and to DNA depletion, inhibition of β-oxidation and depletion of Coenzyme A are further metabolic mechanisms that contribute to liver injury in DILI [88,103,111]. In phase II of biotransformation, reactive metabolites are detoxified by conjugation with chemical groups like glutathione, glucuronate or acetyl. This metabolic step increases hydrophilicity of the drug metabolite making excretion into bile or urine possible. Genetic variations of enzymes involved in these metabolic steps, such as glutathione *S*-transferase (GST), have been associated with DILI/HDS [86]. As an example, polymorphisms of *N*-acetyltransferase 2 (NAT2) result in slow acetylation and increase susceptibility to isoniazide-induced DILI [112]. The consequences of impaired biotransformation, oxidative stress and consumed protective mechanisms are cell death, apoptosis and necrosis [113]. Damage-associated molecular pattern molecules (DAMPs) are involved in these processes and perpetuate liver damage in DILI/HDS [114].

Hepatobiliary excretion is one of the last steps of drug metabolism. The biliary transporter multidrug resistance protein 3 (MDR3) is a phospholipid flippase located in the canalicular membrane of hepatocytes. It mediates the biliary excretion of phosphatidylcholine. Phosphatidylcholine promotes the formation of micelles and thereby prevents damage of the biliary epithelium by bile acids. MDR3 activity is inhibited by antifungal and antipsychotic drugs [115,116]. Impaired hepatobiliary excretion could be addressed therapeutically, especially for cholestatic DILI/HDS. Therefore, lessons can be learned from new drugs for cholestatic autoimmune diseases such as primary biliary cholangitis (PBC). New drugs for PBC are pluripotent and aim at diverse mechanisms that are involved in bile acid metabolism and circulation, e.g., induction of excretory bile acid pumps. Obeticholic acid is a farnesoid X receptor (FXR)-agonist and is approved for second-line therapy of PBC [117]. Among other effects, FXR upregulates the bile salt export pump (BSEP). Thereby, FXR-agonists might ameliorate cholestatic DILI/HDS that are caused by drugs inhibiting BSEP, such as imatinib [115].

Molecular mechanisms that have been analysed in HDS particularly involve checkpoints of metabolism. Several herbs contain pyrrolizidine alkaloids that can induce sinusoidal obstruction syndrome [118,119]. On a molecular level, toxic metabolites of pyrrolizidine alkaloids promote endothelial damage. Germander is another example of herbal-induced liver injury for which the mechanistic background is known. Its reactive metabolites (diterpenoids) deplete hepatic glutathione levels and induce apoptosis of hepatocytes [120,121]. Green tea extracts such as catechins are suspected to cause a relevant number of cases of idiosyncratic HDS [122]. However, the molecular mechanism beyond liver damage induced by green tea extracts is not clear.

### 3.4. Pro-Inflammatory Mechanisms

Drug-specific T cells have been identified in DILI/HDS. CD8+ T cells have been detected in peripheral blood of patients with flucloxacillin- and amoxicillin-clavulanate-induced DILI [123,124]. Peripheral blood mononuclear cells (PBMC) from patients with amoxicillin-clavulanate-induced DILI proliferated and expressed the pro-inflammatory cytokine interferon gamma (IFNγ) in co-culture with amoxicillin-clavulanate [124]. IFNγ is one of the main mediators of tissue damage in many forms of acute hepatitis. It stimulates Kupffer cells and enhances expression of HLA class I and II on hepatocytes. Detection of circulating drug-specific T cells has been used for diagnostic purposes by the so-called lymphocyte transformation test [125]. Nonetheless, its diagnostic impact is limited due to restricted standardization and reproducibility. After all, drug-specific T cells have been detected in only about 50% of DILI cases.

Mouse models gave insight into pro-inflammatory mechanisms contributing to DILI/HDS. In addition to impaired metabolic pathways, pro-inflammatory immune reactions have been analysed in models of APAP-induced liver injury [12]. Knockouts of pro- or anti-inflammatory cytokines decreased or increased the susceptibility to APAP-induced DILI. Besides, APAP mouse models support a linkage between drug metabolism and immune response: glutathione depletion made hepatocytes more susceptible to TNFα-mediated apoptosis [126]. Pro-inflammatory Th17 cells have been detected in a murine model of d-penicillamine-induced “autoimmune-like” adverse reaction [127].

A thorough characterization of peripheral and intrahepatic drug-specific T cells in human DILI/HDS is pending. Cytokine profiling seems to support an involvement of IL-17 [128]. Intrahepatic analyses of infiltrating and resident immune competent cells have been performed in human DILI/HDS, but only in a limited manner [129]. These analyses are of importance since PBMC do not always reflect the local conditions. Full characterization of intrahepatic immune competent cells could offer specific immuno-modulating treatment options for DILI/HDS, such as blockage of pro-inflammatory cytokines. However, immunomodulation bears some risks in DILI/HDS. Cytokine-blockage by biologicals like anti-TNFα has been associated with the induction of “immune-mediated” DILI mimicking AIH [130]. Checkpoint-inhibitors like nivolomab and pembrolizumab, targeting programmed cell death-1 (PD-1), or ipilimumab, blocking CTLA-4, can induce adverse autoimmune reactions like hepatitis, colitis, rash, endocrinopathies and pneumonitis [131]. Attempts to specifically block pro-inflammatory pathways have been made for AIH in small case series. Infliximab has been applied successfully as a second-line therapy for difficult-to-treat AIH patients [132]. Rituximab (anti-CD20) has also been used as a rescue therapy for AIH [133]. However, these immunomodulating therapies for AIH are not well-grounded on mechanistic findings.

For the majority of AIH patients, the main antigen is unknown. Therefore, pro-inflammatory mechanisms have been analysed in a more general and not antigen-specific way in the past. It is not clear which kind of T cells response (CD4+, Th1, Th2, CD8+, Th17 or γδT cells) is predominating in human AIH or its mouse models [134,135,136,137,138,139,140]. Antigen-specific analyses have been performed in the subgroups of anti-SLA/LP+ or anti-LKM+ AIH patients. In such an antigen-specific approach, T cell responses were polyclonal, B- and T cell epitopes overlapped and distinct epitopes induced distinct cytokines [141]. An update on the prevailing pro-inflammatory cell type and identification of the main antigen for the majority of AIH patients are two issues on the current research agenda for AIH [142].

In addition to immune competent cells and cytokines, microRNAs (miRNAs) also seem to be involved in molecular mechanisms of DILI/HDS. MiRNAs are small, non-coding RNAs regulating diverse biological processes including inflammation and apoptosis [143]. Due to their organ specificity, their role as biomarkers for liver damage has been investigated in the past [144]. miRNA include various molecules with different biological functions [145]: some convey liver damage [146], others adopt regulatory and anti-inflammatory functions in murine models of DILI [147,148]. The role of miRNAs for AIH has not been investigated yet.

### 3.5. Regulatory Mechanisms

Regulatory T cells (Treg) are the main cellular mediators of immune tolerance. Their impairment is associated with various immune-mediated and autoimmune diseases [149]. By the expression of regulatory cytokines (such as TGFβ, IL-10, etc.) or cell-cell-contacts, Treg are able to suppress pro-inflammatory effector cells. Mouse models of AIH suggest a relevant role of Treg in its pathogenesis. In a model that combines the loss of Treg with the knockout of PD-1 (a suppressor of T cell activity), spontaneous fulminant hepatitis results [150]. Consequently, the role of Treg has been investigated in human AIH and a loss of Treg numbers and an impaired Treg function have been proposed [151,152]. So far, these results could not be confirmed by others [153,154]. After all, it is unclear why regulatory mechanisms fail to control inflammation in times of pronounced activity or during a relapse of AIH. In times of spontaneous remission, regulatory mechanisms seem to be restored.

“Immune-mediated”, idiosyncratic DILI/HDS might be, at least partly, a result of impaired immune regulation. However, the contribution of Treg is not clear. Other regulatory mechanisms than Treg-mediated tolerance have been investigated in DILI/HDS. In a model of amodiaquine-induced DILI, mice with a knockout for Cbl-b (Casitas B-cell lymphoma), an E3 ubiquitin-protein ligase that negatively regulates T cell activity, or a knockout for PD-1 developed more severe liver injury after drug exposure than wildtype mice [155]. However, adaptation and restoration of tolerance occurred in the further course. Additional blockage of CTLA-4 was able to break regulatory mechanisms again. This illustrates that a single defect of regulatory checkpoints is rarely sufficient to disrupt hepatic tolerance in the long run. Defects of the regulatory IL-10 signalling pathway have been associated with DILI as well [80]. Promoter variants and reduced IL-10 expression have been detected in DILI patients and were associated with worse clinical outcome [156]. The protective role of Th22 cells has been investigated in DILI patients: an increase of peripheral and intrahepatic IL-22-secreting T cells was associated with improved liver regeneration [157].

The role of eosinophilia in blood and livers of DILI/HDS deserves more attention in future studies: it is unclear whether their presence is an unspecific epiphenomenon or whether they mediate specific pro-inflammatory or even regulatory signals. Low levels of peripheral eosinophils have been associated with a worse clinical outcome in DILI patients [158]. Recently, a protective role of eosinophils for DILI has been supported by intrahepatic analyses [2].

In mild forms of DILI/HDS, drug intake results in slight elevation of liver enzymes returning to normal levels spontaneously during continuation of the drug. This kind of adaptation is probably the result of restored regulatory mechanisms. Adaptation has been taken advantage for drugs that cannot be replaced easily and have to be continued with acceptable risks. For these drugs, re-challenge after the first episode of liver injury can be considered. Such an approach has been applied successfully for tuberculostatic drugs, for instance [159]. However, adaptation is unpredictable and re-challenge always bears the risk of severe recurrence of liver injury.

## 4. Summary

Some forms of DILI/HDS can adapt clinical characteristics of AIH, such as the presence of autoantibodies and infiltration of the liver by immune competent cells. Definition and nomenclature of this “autoimmune(-like)” subgroup of DILI/HDS is not standardized. Differentiation between AIH and “autoimmune(-like)” DILI/HDS is not easy. Nonetheless, the correct diagnosis is important since AIH requires long-term immunosuppression, whereas DILI/HDS does not. Cases of misdiagnosis, incorrect treatment and insufficient surveillance have certainly been the consequence of this mix-up in the past.

Molecular mechanisms have not been analysed in the subgroup of “autoimmune(-like)” DILI/HDS yet. However, several pathogenetic mechanisms are shared by classical DILI/HDS and AIH. They hint at potential molecular processes of “autoimmune(-like)” DILI/HDS. Genetic associations with HLA-variants point to a relevant role of antigen presentation for both DILI/HDS and AIH. Indirectly, these associations suggest a role of T effector cells in conveying hepatic inflammation in both entities. Still, a thorough characterization of intrahepatic, pro-inflammatory immune competent cells is pending for both DILI/HDS and AIH. In addition to pro-inflammatory mechanisms, molecular and cellular representatives of tolerance, such as regulatory T cells, could be impaired in “autoimmune(-like)” DILI/HDS and deserve further research.

Currently, in comparison to AIH, there is one big advantage for the investigation of molecular mechanisms of DILI/HDS in general and of “autoimmune(-like)” DILI/HDS: the main antigen is known for DILI/HDS (the drug, a metabolite of the drug or a drug/metabolite-carrier adduct), but not for AIH. The relevant question for DILI/HDS in general remains which dysregulated molecular mechanisms render an individual susceptible to DILI/HDS. Possibly, not a single defect, but instead a combination of defects increases susceptibility. Therefore, the whole cascade of mechanisms needs to be analysed for a single drug, including checkpoints like metabolism, neoantigen formation, haptenization, antigen presentation, co-stimulation and co-regulation as well as pro- and anti-inflammatory mechanisms. Due to shared molecular checkpoints of DILI/HDS and AIH, deeper pathomechanistic insights into one entity can improve the understanding of the other and can promote more target-oriented treatment. In the past, the research fields of DILI/HDS and primary autoimmune liver diseases like AIH have mostly been worked on separate from one another. But as lessons can be learned from each other, especially with regard to “autoimmune(-like)” DILI/HDS, scientific exchange must be encouraged.

## Figures and Tables

**Table 1:** Clinical scenarios involving both DILI/HDS and AIH.

Scenario	Characteristics
DILI/HDS on top of AIH	◾ Can be misinterpreted as an aggressive course of AIH if the causative agent is not identified ◾ In most cases of AIH, IgG/gammaglobulins parallel the increase of transaminases; this helps to differentiate AIH from DILI/HDS
Drug-induced AIH	◾ Drug intake triggering the chronic course of AIH is an unlikely scenario ◾ Coincidence of drug intake and pre-existing AIH seems more likely
Second episode of DILI mimics relapsing course of AIH	◾ According to current studies, this scenario is rare ◾ Repeated drug history is helpful to identify the causative agents
Chronic DILI mimics AIH	◾ Chronic DILI/HDS through sustained intake of the causative drug is possible ◾ Underreporting (especially of analgetics, HDS, etc.) hampers identification of the causative drug ◾ However, the presence of cirrhosis favours the diagnosis of AIH and makes DILI/HDS less likely
DILI/HDS with characteristics of AIH (“autoimmune(-like)” DILI/HDS, “immune-mediated” DILI/HDS)	◾ The terms are used for DILI/HDS cases characterized by the presence of autoantibodies and/or infiltration of the liver by immune competent cells ◾ However, most of the autoantibodies (e.g., ANA and anti-SMA) are not disease-specific ◾ Demarcation of “autoimmune(-like)” DILI/HDS from AIH is difficult ◾ Features supporting the diagnosis of AIH are a relapse of transaminases and IgG/gammaglobulins after steroid withdrawal and a chronic, fluctuating course ◾ Close monitoring of transaminases (weekly for the first 1–2 months, every 2–3 weeks for the next 2–3 months, every 3 months for the next 1–2 years) and IgG/gammaglobulins is necessary to confirm the correct diagnosis

Abbreviations: DILI/HDS: Herbal and Dietary Supplements; AIH: Autoimmune Hepatitis; ANA: anti-nuclear antibodies; SMA smooth muscle antigen.

**Table 2:** Molecular mechanisms of DILI/HDS and AIH.

Mechanism	Characteristics
Antigen presentation	◾ Genetic associations with HLA-(human leucocyte antigen) variants hint at a relevant role of antigen presentation for both DILI/HDS and AIH (e.g., HLA-DRB1*03:01 and DRB1*04:01 are associated with the risk for AIH and HLA-B*57:01 is associated with the risk for flucloxacillin-induced DILI) ◾ In DILI/HDS, neoantigen formation and haptenization seem to be involved, but have been investigated in only few cases (e.g., in flucloxacillin-induced DILI) ◾ The hepatic microenvironment has not been characterized thoroughly for DILI/HDS yet (different kinds of antigen presenting cells, costimulatory molecules, etc.) ◾ For the majority of AIH patients, the main antigen is unknown. In 5-10% of AIH patients, disease-specific antibodies (anti-SLPA/LP, anti-LKM and anti-LC1) are detected for which the respective antigens have been characterized (SEPSECS, CYP, 2D6 and FTCD). These antigens could be involved in the pathogenesis of AIH.
Metabolism	◾ Drug- and host-specific factors influence metabolism and susceptibility to DILI/HDS (lipophilicity, dosage, age, sex, ongoing inflammation etc.). ◾ Genetic variants of checkpoints of phase I and II biotransformation increase the risk for DILI/HDS (e.g., polymorphisms of NAT2 have been associated with the risk for isoniazide-induced DILI) ◾ New treatments for cholestatic liver diseases like PBC might offer therapeutic options for impaired hepatobiliary excretion of drugs in DILI/HDS
Pro-inflammatory mechanisms	◾ In flucloxacillin- and amoxicillin-clavulanate-induced DILI, peripheral effector cells have been characterized and IFNγ has been identified as a relevant proinflammatory cytokine. ◾ In AIH, conflicting results exist about the main proinflammatory cell type (CD4+, Th1, Th2, CD8+, Th17 or γδT cells) ◾ A full characterization of the composition of pro-inflammatory immune competent cells and effector cytokines is pending, both for DILI/HDS and AIH. Intrahepatic analyses are required, since peripheral blood cells probably not reflect the situation in the liver ◾ Identification of relevant pro-inflammatory pathways can offer specific treatment options for both DILI/HDS and AIH
Regulatory mechanisms	◾ An impairment of regulatory mechanisms has been proposed for several inflammatory liver diseases ◾ Mediators of tolerance are, among others, regulatory T cells (Treg) and anti-inflammatory cytokines like IL-10 or TGFβ ◾ Restoration of tolerance could be a therapeutic aim for both DILI/HDS and AIH ◾ In mild forms of DILI, transient elevation of liver enzymes returning to normal levels spontaneously might represent restoration of tolerance. The molecular mechanisms for these clinical observations have not yet been analyzed

SLPA/LP: soluble liver antigen/liver-pancreas antigen; LKM: liver-kidney microsomes; LC1: liver cytosol 1; SEPSECS: *O*-phosphoseryl-tRNA:selenocysteinyl-tRNA synthase; CYP: cytochrome P450; FTCD: formiminotransferase cyclodeaminase; NAT2: *N*-acetyltransferase 2; IFNγ: interferon gamma; IL: interleukin; TGFβ: transforming growth factor beta.

## Abbreviations

AIH	Autoimmune hepatitis
ALH	Acute liver failure
ANA	Anti-nuclear antibodies
APAP	(*N*-) Acetyl-para-aminophenol, acetaminophen
APC	Antigen-presenting cells
CARD10	Caspase recruitment domain 10
Cbl-bCTLA-4	Casitas B-cell lymphomaCytotoxic T lymphocyte antigen 4
CYP	Cytochrome P450
DAMPs	Damage associated molecular pattern molecules
DILI	Drug-induced liver injury
DC	Dendritic cells
DRESS	Drug rash with eosinophilia and systemic symptoms
ELISAFTCD	Enzyme-linked Immunosorbent AssayFormiminotransferase cyclodeaminase
FXR	Farnesoid X receptor
GWAS	Genome-wide association study
GST	Glutathione *S*-transferase
HDS	Herbal and dietary supplements
HLA	Human leucocyte antigen
IAIHG	International autoimmune hepatitis group
IL	Interleukin
IFNγ	Interferon gamma
LC1	Liver cytosol 1
LKM	Liver-kidney microsomes
LSEC	Liver sinusoidal endothelial cells
MELD	Model of end-stage liver disease
MDR3	Multidrug resistance protein 3
MHC	Major histocompatibility complex
MS	Multiple sclerosis
NAC	*N*-acetylcysteine
NAPQI	*N*-acetyl-*p*-benzoquinoneimine
NAT2	*N*-acetyltransferase 2
PBMC	Peripheral blood mononuclear cells
PD-1	Programmed cell death-1
RNA	Ribonucleic acid
ROS	Reactive oxygen species
RUCAM	Roussel uclaf causality assessment method
SEPSECS	*O*-phosphoseryl-tRNA:selenocysteinyl-tRNA synthase
SH2B3	Scr homology 2 adaptor protein 3
SLA/LP	Soluble liver antigen/Liver-pancreas antigen
SMA	Smooth muscle antigen
TGFβ	Transforming growth factor beta
TNFα	Tumor necrosis factor-alpha
Treg	Regulatory T cells
